# Editorial: Emerging sustainable and green technologies for improving agricultural production

**DOI:** 10.3389/fpls.2025.1678701

**Published:** 2025-08-28

**Authors:** Ruonan Ma, Hangbo Xu, Libin Zhou, Gyungsoon Park, Zhen Jiao

**Affiliations:** ^1^ Zhengzhou Research Base, National Key Laboratory of Cotton Bio-breeding and Integrated Utilization, School of Agricultural Sciences, Zhengzhou University, Zhengzhou, China; ^2^ Henan Key Laboratory of Ion-beam Green Agriculture Bioengineering, Zhengzhou University, Zhengzhou, China; ^3^ Institute of Modern Physics, Chinese Academy of Sciences, Lanzhou, China; ^4^ Plasma Bioscience Research Center, Department of Plasma-Bio Display, Kwangwoon University, Seoul, Republic of Korea

**Keywords:** computer vision, atmospheric cold plasma, ion beams, nanoparticles, plant phenotype, plant growth, crop yield, plant pathogen

## Introduction

Nowadays, the world is facing food shortages with the increasing global population, decreasing food sources, and deteriorating environment. The traditional methods (e.g. seed priming, stratification, scarification, ultrasound, disinfectants, fungicides, hormones, and fertilizers) have been employed to improve agricultural production. However, most of these methods have their own limitations. To address these challenges, this Research Topic “Emerging sustainable and green technologies for improving agricultural production” aims to apply computer vision technology, emerging physical technologies (Ion beam irradiation, low-temperature plasma, magnetic field, and micro-nano bubble), nanotechnology and intelligent filed management for precise analysis of plant phenotype, efficient control of plant disease, regulation of seed germination and plant growth, and improvement of crop yield. During this period, a total of 41 papers were submitted to the Research Topic and 23 papers from 156 authors were published.

## The application of computer vision technology in agriculture

Monitoring the plant growth phenotype has great significance for promoting the efficient and precise implementation of agronomic operations, screening superior traits and breeding new varieties. Traditional analytical techniques mainly rely on manual identification, which involves high labor intensity, high labor costs, and is prone to subjective errors. In recent years, with the development of deep learning technology, computer vision has become an important tool for plant phenotypic analysis. The YOLO (You only look once) series of algorithms feature fast detection speed, high efficiency and high precision, and have demonstrated extremely high application value in agriculture.

For example, the prevalent measurement methods for corn seeds are traditional, consuming substantial process resources. Yu et al. proposed an enhanced YOLOv8 target detection model, EBS - YOLOv8, for detecting corn seed germination. They demonstrated this method for germination potential put forward in this paper can effectively depict the rate variation of seeds during the germination process, thus offering a novel perspective for future research on seed germination potential. Accurate and rapid identification of cabbage posture is crucial for minimizing damage to cabbage heads during mechanical harvesting. Shen et al. introduced YOLOv5-POS, an innovative cabbage posture prediction approach. Cabbage posture recognition was completed within 28 milliseconds, enabling real-time harvesting. This method provided a highly accurate and efficient solution for automated harvesting, minimized crop damage, and improved operational efficiency. Identifying grape bunches is crucial for maintaining the quality and quantity of grapes, as well as managing pests and diseases. Yang et al. proposes a lightweight detection method named YOLOv8s-grape. The thesis proposes a lightweight and efficient model for grape bunch detection and biophysical anomaly assessment in complex environments based on YOLOv8 by redesigning the network structure. Green pepper fruits have colors similar to leaves and are often occluded by each other, posing challenges for detection. Du et al. proposed an automatic counting method for green pepper fruits based on object detection and multi-object tracking algorithm. The above researched indicated that not only enhanced the efficiency and precision of agricultural production by YOLO, but also promoted the development of intelligent agriculture.

## The application of physical methods for promoting plant growth

In the face of the food crisis, the application of physical technologies to increase food production becomes particularly significant. Ion beam irradiation technology, low-temperature plasma technology, magnetic field technology, and micro-nano bubble technology, etc. are attracting more and more attention from researchers.

Plasma-activated water (PAW) can enhance seed germination, growth, and biomass production. It is important to explore PAW’s potential in improving the productivity of sorghum and possibly other crops. Beak et al. investigated the effects of PAW irrigation on young sorghum seedlings through phenotypic and transcriptional analyses. Not only enhances sorghum seedling growth through transcriptional regulation but also has the potential to optimize agricultural practices by increasing crop yield. In addition, Veerana et al. found that plasma enhanced growth and salinity tolerance of bok choy (*Brassica rapa* subsp. chinensis) in hydroponic culture. Ion beam irradiation technology is often used in mutagenic breeding. However, in recent years, its contemporary stimulating effect on seeds has also been discovered, such as promoting crop growth and enhancing stress resistance, etc. The Arabidopsis seeds were irradiated by ^12^C^6+^. Yin et al. demonstrated that 170 DEGs were present in the 50 Gy and 200 Gy groups and GO enrichment indicated that they were mainly associated with stress resistance and cell wall homeostasis. Low-dose heavy ion beam irradiation induces ROS production in plants, thereby accelerating seedling growth, while high-dose irradiation leads to the accumulation of excess ROS and thereby severely inhibits plant growth. Magnetoelectric activated water can significantly improves soil salt leaching and water use efficiency, demonstrating positive effects on enhancing soil water retention, promoting crop growth, and increasing yields. Lei et al. clarify the effects of coupling improvement agents under magnetoelectric activated water irrigation conditions on the photosynthetic physiological traits, grain nutrients, and yield of spring maize in the arid region of northwest China. Microbubbles are referred as bubbles with a diameter less than 100 microns and situated between micron- and nanoscale dimensions. They can generate through various mechanisms, such as rotational shear, pressure dissolution, electrochemistry, micropore pressure and mixed jet flow. These bubbles possessed unique physical and chemical properties, including slow bubble rise in solution, prolonged residence time, enhanced solubility, expanded specific surface area, accelerated gas−liquid mass transfer rate, elevated interface point, and spontaneous generation of free radicals. Moreover, it can also increase the ozone saturation concentration in water, leading to its decomposition into oxygen for crop growth and development, thereby mitigating hypoxia-related issues in crops. In the study of Zhao et al., the micro/nano bubble technology was applied to achieve a saturation state of bubble nutrient solution, including micro-nano oxygen (O_2_ group) and micro-nano ozone (O_3_ group) bubble nutrient solutions. The application of micro/nano (O_2_ and O_3_) bubble nutrient solutions to substrate-cultured lettuce plants increased the amount of dissolved oxygen in the nutrient solution, increased the lettuce yield, and elevated the net photosynthetic rate, conductance of H_2_O and intercellular carbon dioxide concentration of lettuce plants.

## The application of nanomaterials in agriculture

In the pursuit of sustainable development, nanotechnology provides effective solutions for enhancing agricultural productivity. Nanomaterials (NMs) can be effective in increasing plant abiotic and biotic stress tolerance. Trzcińska-Wencel et al. aimed to evaluate the effects of biologically synthesized silver nanoparticles (AgNPs) from *Fusarium solani* IOR 825 on the growth of Zea mays. They demonstrated the lowest tested concentration of AgNPs (32 µg mL^−1^) on maize efficiently inhibits maize-borne pathogens, without any negative effect on plant growth and chlorophyll content. Moreover, it does not provoke oxidative stress. Nonetheless, various nanoparticles can influence plant growth in diverse manners, often through distinct mechanisms of action. Beyond their direct effects on the plant itself, they frequently alter the physicochemical properties of the soil and modulate the structure of microbial communities in the rhizosphere. Zhang et al. reviewed the multifaceted impacts of nanoparticles on plant nutrient absorption and soil microbial communities. Nanoparticles enhance plant growth and development by facilitating nutrient uptake, modulating rhizosphere microorganisms, and enhancing soil physiochemical properties, among other benefits, with a focus on their positive effects on plants.

## Intelligent field management

Straw return is regarded as a widely used field management strategy for improving soil health, but its comprehensive effect on crop grain yield and quality remains elusive. Zhang et al. reviewed straw return enhances grain yield and quality of three main crops. A meta-analysis containing 1822 pairs of observations from 78 studies was conducted to quantify the effect of straw return on grain yield and quality of three main crops (maize, rice, and wheat). On average, compared with no straw return, straw return significantly (p < 0.05) increased grain yield (+4.3%), protein content (+2.5%), total amino acids concentration (+1.2%), and grain phosphorus content (+3.6%), respectively. The effects of distinct straw retention modes on soil denitrification activity have rarely been discriminated and the underlying mechanisms remain unclear. Zhang et al. coupled field and incubation experiments to explore the characteristics of soil denitrification activity, soil and standing water physicochemical properties, and the abundance, community diversity, and co-occurrence network of nosZ denitrifiers, based on a paddy field implementing 10-year straw retention under a rice–wheat rotation system.

Wheat harvesting is highly time-sensitive, with the optimal period for harvesting being very short. The scheduling of agricultural machinery is a crucial component of modern agriculture and is closely related to the productivity of agricultural operations. In response to the issue of harvesting machine failures affecting crop harvesting timing, Liu et al. develops an emergency scheduling model and proposes a hybrid optimization algorithm that combines a genetic algorithm and an ant colony algorithm. As a renewable forest resource, bamboo plays a role in sustainable forest development. Recently, a strip clear-cutting (StC) was theoretically proposed to promote the sustainability of bamboo production, Liang et al. verified that StC for Phyllostachys glauca forests is feasible and sustainable as its sustainability index outweighs those of traditional cutting systems (SeC and ClC), and 10 m is the optimum distance for the strip width of StC.

In conclusion, the integrated application of computer vision, physical intervention, nanomaterials and intelligent management is driving agriculture to innovate in the direction of high efficiency, precision and sustainability, providing multi-dimensional solutions for addressing food security challenges.

